# Science of science: A multidisciplinary field studying science

**DOI:** 10.1016/j.heliyon.2024.e36066

**Published:** 2024-08-13

**Authors:** Alexander Krauss

**Affiliations:** a London School of Economics, London, UK; b Institute for Economic Analysis, Spanish National Research Council, Barcelona, Spain

**Keywords:** Science of science, Metascience, Foundations of science, Foundations of knowledge, Limits of science, Limits of knowledge, Origins of science, Origins of knowledge

## Abstract

Science and knowledge are studied by researchers across many disciplines, examining how they are developed, what their current boundaries are and how we can advance them. By integrating evidence across disparate disciplines, the holistic field of *science of science* can address these foundational questions. This field illustrates how science is shaped by many interconnected factors: the cognitive processes of scientists, the historical evolution of science, economic incentives, institutional influences, computational approaches, statistical, mathematical and instrumental foundations of scientific inference, scientometric measures, philosophical and ethical dimensions of scientific concepts, among other influences. Achieving a comprehensive overview of a multifaceted field like the *science of science* requires pulling together evidence from the many sub-fields studying science across the natural and social sciences and humanities. This enables developing an interdisciplinary perspective of scientific practice, a more holistic understanding of scientific processes and outcomes, and more nuanced perspectives to how scientific research is conducted, influenced and evolves. It enables leveraging the strengths of various disciplines to create a holistic view of the foundations of science. Different researchers study science from their own disciplinary perspective and use their own methods, and there is a large divide between quantitative and qualitative researchers as they commonly do not read or cite research using other methodological approaches. A broader, synthesizing paper employing a qualitative approach can however help provide a bridge between disciplines by pulling together aspects of science (economic, scientometric, psychological, philosophical etc.). Such an approach enables identifying, across the range of fields, the powerful role of our scientific methods and instruments in shaping most aspects of our knowledge and science, whereas economic, social and historical influences help shape what knowledge we pursue. A unifying theory is then outlined for science of science – the *new-methods-drive-science* theory.

## 1 Introduction

Science shapes nearly all aspects of our lives – from the medicine we take to the technology we use. Despite the enormous importance of science on our lives, we still do not understand well why science evolved the way it did, what its present limits are and how to push those limits. Scientists do not generally have the time to step back and study science itself. In attempting to address these questions, most existing studies that do exist take one individual disciplinary perspective – studying only the history of science [[1], [2], [3]], scientometrics [[4], [5], [6], [7], [8], [9]], sociology of science [[10], [11], [12], [13]], philosophy of science [[14], [15], [16], [17], [18], [19]] or psychology of science [[20], [21], [22], [23], [24]]. But we are not able to understand science, a multidimensional phenomenon, from just one disciplinary perspective.

The field of *science of science* can overcome this challenge by bringing together disparate evidence across many sub-fields studying science. It assesses the cognitive processes of scientists involved in generating and accepting scientific knowledge by integrating evidence from cognitive science of science. It examines team collaborations, career dynamics and networks of scientific communities by evaluating data from scientometrics and network science. It studies the socioeconomic context and economic incentives shaping the direction of scientific practices and institutions by including evidence from economics of science and sociology of science. It traces the historical evolution of science and its methodologies by combining insights from history of science and methodology of science. It investigates the computational approaches that generate scientific knowledge by integrating evidence from computer science of science. It evaluates the statistical and mathematical foundations of scientific inference and reasoning by incorporating insights from statistics and mathematics of science. It studies the philosophical and ethical aspects of scientific assumptions and concepts by building in insights from philosophy of science. Together, the range of evidence collectively contributes to a more nuanced and multidisciplinary understanding of the interplay of factors influencing scientific knowledge and science.

Combining evidence and insights from diverse disciplines across the science of science thus offers an interdisciplinary perspective on scientific practices, a more holistic understanding of scientific processes and outcomes, and more nuanced perspectives to how scientific research is conducted, influenced and evolves.

Comprehending the environment and climate change for instance hinges on the synthesis of methodologies and data across disciplines such as ecology, physics, chemistry, physical geography, natural resource management, economics, and atmospheric science. Unravelling the complexities of our human body requires the combination of methodologies and data from biomedicine, genetics, physiology, epidemiology, neuroscience, nutrition science and biostatistics. An interdisciplinary approach is also the only way we can attain a comprehensive understanding of a system as multifaceted as science. Given the absence of an integrated approach, our comprehension of science lags behind our comprehension of phenomena like the environment, the human body and other complex phenomena. A holistic comprehension is best achieved by leveraging diverse methodologies from disparate fields that grounds our knowledge in those disparate strands of evidence.

The methods used to study science and the most studied and important feature of science strongly diverge across the disconnected disciplines. Scientometricians and network scientists focus on citations and publication records [4,5], while sociologists of science on social influences on science [10,11]. Economists highlight the role of funding and incentive structures for rewarding and advancing science [[25], [26], [27], [28]], while statisticians emphasise the methodological constraints and biases in designing and improving studies [29,30]. Historians and philosophers of science concentrate on scientific theories [1,14,15,18,31], and so on. Despite the significance of these individual aspects, there remains a lack of integration into a unified account.

The historian of science Thomas Kuhn argued that science goes through paradigm shifts, which are foundational changes in scientific theories, and thus science is not cumulative. His account of science has been highly influential but no consensus yet exists on how science progresses [1,32]. In recent articles in *Science* and *Nature*, scientists studying science commonly adopt the perspective of scientometrics which is the field that studies science by analysing and measuring aspects of scientific publications and citations commonly using large-scale data. Scientometricians including network scientists investigate various features such as career trajectories, team collaboration, research output, and networks of scientists and institutions [4,5,8,9,[33], [34], [35], [36]]. This big-data perspective has produced explanations about the dynamics of science. But it has not yet provided an explanation of the common origins, boundaries and driving forces of science and their methods. These researchers acknowledge that the field's success ‘depends on us overcoming traditional disciplinary barriers’ and acknowledge the limitations of scientometric methods to study how science works: ‘this bias toward citations is reflective of the current landscape of the field, [and] it highlights the need to go beyond citations as the only “currency” of science’ [4]. ^cf^ [33,35,37].

Existing research, largely confined within disciplinary boundaries [35], has resulted in blind spots and gaps in our understanding of science. Here we outline a way to reduce the gaps to this debate that, to date, largely occurs within separate (not across) fields. Factors emphasised as highly important in a given field end up being much less important once compared with the range of factors across all fields, which has implications for shifting the research focus in different fields and how we understand the driving forces of science.

While specialised fields have many advantages, a meta-approach is also indispensable for synthesizing fragmented knowledge. Only through such integration can we discern what factors are most and least important in understanding science, shift more attention towards them and uncover a common mechanism across fields. This enables overcoming the common approach in the literature of each field proposing explanations of science and what drives it, but independent of the explanations in neighbouring fields. Here we aim to expand *science of science* by integrating the subfields mentioned above and others that study science and have remained isolated to date – from methodology of science [30,38,39], economics of science [[25], [26], [27], [28],^40^] and computer science of science [[41], [42], [43]], to cognitive science of science [[44], [45], [46], [47]], biology of science [[48], [49], [50], [51]], anthropology of science [[52], [53], [54], [55], [56]] and archaeology of science [[57], [58], [59]]. ^cf^ [4,5,33]. The aim here is that science of science will emerge as a unified field that integrates the range of subfields studying science, similar to the environmental sciences and medical sciences that are unified fields that have tackled previously fragmented areas of knowledge.

A central argument here is that to understand science comprehensively we need to integrate evidence of the abilities and conditions that have enabled developing science (biological, cognitive, social and methodological), the abilities and conditions shaping the scope of science (including, in addition, historical, economic etc.) and, most importantly, the abilities and conditions allowing us to expand the present limits of science (mainly methodological but also cognitive, sensory and social). The account of science offered here explains how our scientific methods and instruments play a pivotal role in shaping the foundations and constraints of science by determining how we observe, measure and experiment, namely how we do science. The role and importance of other factors that can influence science (from funding and incentives to the scientific community) vary widely: in groundbreaking scientific publications, teams range in size, funding level, age, affiliated university ranking and interdisciplinary nature. With scientific methods being essential for scientific practice and how science is carried out and advanced, a central focus on scientific methods is important to the integration of science of science. Unlike other factors, scientific methods pervade all fields and domains of scientific inquiry and it is a factor that we can directly influence and improve to do science better and foster innovation.

**Fig. 1:**
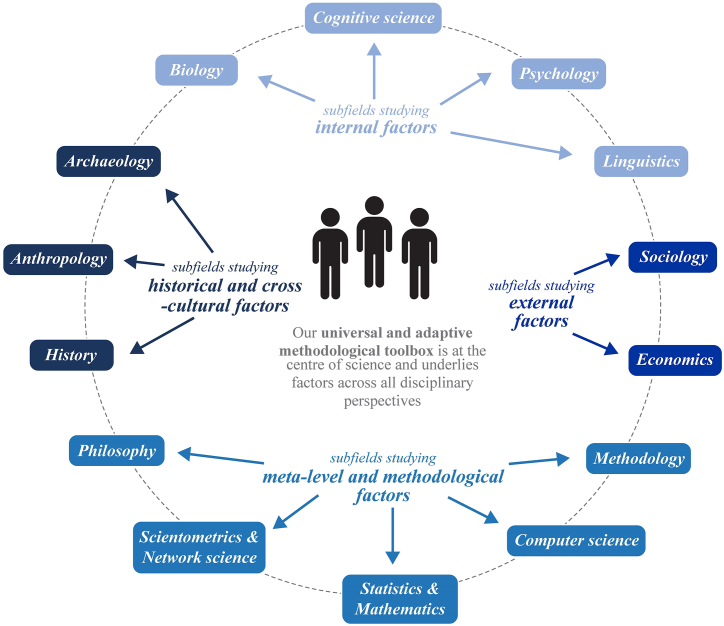
Science of science: explaining the foundations, limits and advancement of science from fourteen subfields – an integrated field that combines multiple methods. Note: Here we demarcate the field of science of science by identifying the fourteen relevant fields that contribute to understanding science and it groups them into four areas. Internal factors refer largely to the human body and mind; external factors refer to our broader environment; historical and cross-cultural factors refer to the past and different contexts; meta-level and methodological factors refer to meta-scientific aspects of science and scientific methodology. Other research domains that can provide insight can be categorised in one of these subfields – e.g. public policy is included in economics of science, and communication sciences in linguistics of science. Finally, cognitive science of science broadly covers our evolved cognitive abilities (observation, abstraction etc.), while psychology of science narrowly covers psychological biases.

Here we will first describe the field in the next section (Section 2) and then outline the integrated field of *science of science* by combining methods and evidence from 14 relevant fields (2.1, 2.2, 2.3, 2.4, 2.5, 2.6, 2.7, 2.8, 2.9, 2.10, 2.11, 2.12, 2.13, 2.14) that are grouped here into four areas – see Fig. 1. These are identified here using the criterion of whether they help explain the origins, foundations or limits of science. Fields like physics and chemistry are not as *directly* relevant and thus not included, as science is a complex system in which we and our methods are at the centre and thus human sciences and methodological fields provide most insight. This holistic and methods-driven account of science is then presented (Section 3). An integrated theory, the *new-methods-drive-science* theory, is outlined that explains how we create new scientific ideas and breakthroughs by developing new methods and instruments. This unifying theory can offer a foundation for the field of science of science (Section 4). We then outline how we can help establish the field. Such breadth (covering 14 different fields) inevitably comes at the cost of less depth on any single field and factor discussed within the paper (i.e. insights from each discipline are simplified to less than a page to fit an article format).

The crux of the argument is that while various subfields studying science each shed light on distinct facets of its evolution and limits, none offers a comprehensive account, but together they can unveil a holistic panorama of science (2.1, 2.2, 2.3, 2.4, 2.5, 2.6, 2.7, 2.8, 2.9, 2.10, 2.11, 2.12, 2.13, 2.14). To achieve integration, a concerted emphasis on scientific methods is warranted, as they constitute the common nexus where disparate subfields converge. Synthesizing these disciplinary perspectives reveals that our methodological toolbox – comprising cutting-edge microscopy, mathematical techniques and x-ray methods – is essential in advancing science by empowering us to perceive, measure and explain the world in novel ways (3, 4). Evidence is derived here from studies using large-scale statistical analysis, experiments, surveys of scientists, historical analysis, big data analysis and other data sources.

While there are many advantages to quantitative papers, there are also cases in which a broader, synthesizing paper that adopts a qualitative approach is needed. To be able to provide a comprehensive overview of a multifaceted field like science of science requires pulling together insights from the diverse sub-fields studying science across the natural, behavioural and social sciences. In doing so, qualitative studies enable exploring complex phenomena and patterns across those diverse disciplines that can be challenging to quantify or replicate precisely. They can contribute to a holistic and nuanced understanding of the different factors influencing scientific processes and outcomes by integrating them together. In the history of science for example, a qualitative approach can help trace the evolution of ideas, methodologies and paradigms. In the sociology of science, it can provide insight into scientists’ motivations and social norms that can influence scientific practices, and the like. Qualitative studies can provide a bridge between disciplines and foster interdisciplinary understanding by pulling together aspects of science (economic, psychological, philosophical etc.). Overall, they can enable us to better understand the broader context influencing the foundations of science and uncover aspects of science that quantitative methods are not designed for. Other related quantitative studies examine the measurable features driving science and discoveries [60,61], and offer statistical data to ground this qualitative research and methodological perspective. Together, qualitative and quantitative studies can contribute to developing a theoretical and conceptual framework that integrates diverse insights into the study of science.

Science is defined here as the study of the natural and social world by using our cognitive abilities (including observation, experimentation and problem solving) and the methods and instruments we develop (including statistics and microscopes) with the aim of describing, explaining, predicting and controlling phenomena.

## 2 Describing science of science

Science of science may initially appear paradoxical – a domain in which scientists engage in science to unravel the complexities of science itself. These researchers conduct the very scientific activity they seek to understand. Methodologists and statisticians scrutinize the constraints and assumptions inherent in scientific methods, which they themselves adopt. Sociologists and psychologists examine the biases and norms shaping scientific research, which can influence their own research. Scientometricians and network scientists assess publications and citation patterns in science by producing publications, which they themselves hope will be cited. Evolutionary biologists explore our cognitive evolution and origins of reasoning, which they themselves have inherited.

**Fig. 2:**
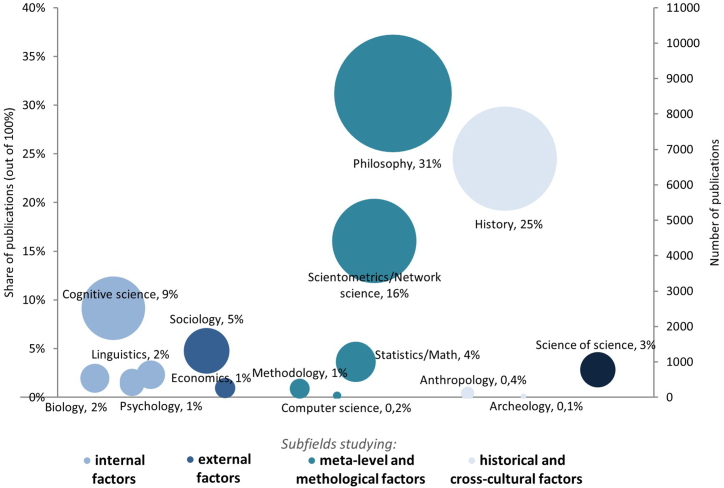
Share and number of publications across the subfields of science of science. Note: Data are derived from Scopus (the largest citation database of scientific journals) and reflect estimates for all existing publications – up to early 2024 – within each subfield [63]. The estimated shares add up to 100 % of publications across all 14 subfields including the 3 % of publications using the term ‘science of science’, ‘metascience’ or ‘metaresearch’ (on the far right). The shares provide a rough estimation of the distribution of research across fields provided in Scopus while they do not capture all publications in each research area. A note in the appendix outlines how the shares are calculated.

Despite the profound societal significance of science and the global cohort of about nine million scientists [62], surprisingly only few are dedicated full-time to studying science. There is a lack of interdisciplinary journals and university departments dedicated to science of science. Across the various subfields studying science, the vast majority of publications cluster in five domains, with the largest concentrations in philosophy of science (31 %), history of science (25 %), scientometrics/network science (16 %), cognitive science of science (9 %) and sociology of science (5 %) (see Fig. 2). Yet this clustering of publications within these five subfields, comprising about 86 % of the total corpus, is largely a consequence of historical contingencies rather than inherent superiority in offering a more comprehensive understanding of the foundations and limits of science than other subfields, like methodology of science and cognitive science of science. Only about 3 % of publications studying science use the term ‘science of science’ (or ‘metascience’ or ‘metaresearch’) (Fig. 2). As an indication of limited integration in the field, very few publications across all subfields use the common terms interdisciplinary, cross-disciplinary or multidisciplinary, at less than 3 % in total. For example, within the over 8000 publications in ‘history of science’, only 3.5 % mention the term ‘inter/cross-/multidisciplinary’ and 0.1 % the term ‘science of science’. Within the over 5000 publications in ‘scientometrics’, only 12 % and 0.6 % mentioned these terms.

Different researchers studying science use a different method and unit of analysis, and thus study different features of science. Disciplinary isolation has led to simplified and at times contradictory views on what feature of science is most important: publications and citations [4,5], paradigm shifts in theories [1,32], the principle of falsification of theories [14,15,31], social practices of scientists [10,11], and so on. Leading researchers have often overinterpreted the particular role of the factor they study compared to other factors, especially the foundational role of our scientific methods and our mind in enabling and constraining science that are not as commonly studied.^(ibid.)^ Classic work in the early origins of science of science goes back at least to Znaniecki in 1923 [64], Ossowska and Ossowski in 1935 [65] and more generally to Galton in 1874 [66] and later de Solla Price in 1963 [67] and Zuckerman in 1977 [68].

In Fig. 2 we outline the landscape of existing research studying science and its concentration in particular subfields. In Fig. 1 we describe the unified approach to the field presented here that is needed to comprehensively understand science, combining the different bodies of research and methods which, to date, have been fragmented from each other.

Within the scientometric community that includes network scientists, the field has however been viewed as the ‘field that relies on big data to unveil the reproducible patterns that govern individual scientific careers and the workings of science’ by studying primarily publications and citations [4]. ^cf.^ [5,33] This common view among scientometricians illustrates how they view the field in terms of one method. Though they recognise that the success of the field ‘depends on us overcoming traditional disciplinary barriers’ [4], there are a range of other disciplinary methods, evidence and perspectives from across the other fields studying science that are not taken into account. Scientometrics has however dominated the research studying science in leading multidisciplinary science journals [4,5,33,35,37]. Fig. 1 outlines what an integrated science of science, without disciplinary divisions, looks like. The integrated field presented here can be defined as follows:

The field of *science of science* is the study of science, and especially the foundations, limits and advancement of science and scientific methods, that integrates methods and evidence from across the natural, behavioural and social sciences.

The field addresses foundational, methodological and meta-scientific aspects of science. It studies fundamental questions that span the scope of the field: What drives science? How do we develop science? What constrains science? How can we improve and advance science? The field assesses the methods and instruments of science, the process of science, how we design, implement and evaluate scientific studies, and domain-specific topics from its subfields. This is done by applying a range of methodologies. These include empirical studies, experiments, surveys of scientists, historical analysis, big data analysis and conceptual analysis, which are integrated across domains and subfields. The field studies science from across disciplinary borders – and both from high-altitude and the bottom-up. The objective of the field is straightforward: by better understanding the foundations and present limits of science and scientific methods, we can do science better and drive new knowledge and discoveries. This is the field of science of science. And we now outline its subfields.

### 2.1 Biology of science

What are the evolutionary origins of science and how can they help us understand science today? Like other animals, our ancestors evolved abilities for vision and other senses that enable us to perceive the world, and evolved other related physiological functions. Species like ours require making observations and acquiring knowledge to be able to survive and meet basic needs. This requires knowing about what foods they can and cannot eat, and about their ecological environment and other animals [57,69]. Chimpanzees for example use different types of tools for extractive foraging. They use stone and wood hammers to crack nuts, sticks to extend their reach and extract termites and honey, and levers for different tasks [21,[70], [71], [72]]. They have a toolkit acquired through learning and experimenting. To use tools requires that they have a clear objective of the tool in mind, predict how it can achieve the objective and understand how it must be applied. This requires understanding the interactions needed between the tool, their hands and the desired outcome. Many animals use tools, from crows to sea otters and octopuses, by manipulating objects for their purposes [70]. Non-human primates also reason about objects, space, quantities and the mental states of others, use classification systems and identify causal relationships [21,70,73].

Our human perspective to the world, as the biological animals we are, also shapes what knowledge and objectives we pursue as we use our mind and the methods we develop to do science. Just by being human and members of our species, we direct more attention to particular phenomena that fall within the environmental niche we have evolved in and live in. Nearly all scientists study aspects of reality relevant to our needs and wants – human biology, human technology, human society, human diseases, human behaviour and other problems and objectives we face. Large funding agencies generally require researchers to outline the human impact of their research to receive funding. Most science funding worldwide is spent on studying human beings, with for example 52 % of public research funding in the US allocated to medicine, life sciences and psychology. The remaining 48 % is allocated to all other disciplines that also generally aim to benefit human beings, including engineering, physical and social sciences, environmental science and computer science [74]. We thus study the world from our anthropocentric context that shapes the scope of science.

### 2.2 Archaeology of science

Archaeological artefacts including sophisticated tools developed by early humans provide evidence into the origin of science and the evolution of our methodological abilities to reason and acquire knowledge and to do science today. We have evolved abilities to observe, solve problems, experiment, categorise, reason causally, and test ideas or hypotheses [22,70]. Using these abilities, early humans such as homo erectus and Neanderthals created complex tools such as hand axes at least about 1.5–2 million years ago [75,76]. Making such tools requires the ability to imagine and plan what they will look like before creating them. Early stone toolmakers needed to make mental representations, inferences and predictions [58,77] – abilities commonly used in contemporary science.

Our species, using these methodological abilities, eventually learned how to domesticate animals that requires knowledge of reproduction, nutritional needs of animals and selective breeding to foster particular traits. We learned how to cultivate crops which requires understanding causal interactions between seeds, rain, soil fertility, erosion and annual cycles. It requires experimenting with seeds, and knowledge about selective planting, storing seeds and often irrigation methods [78].

With the first civilisations, we made large leaps towards science and developed systems of written language and mathematics around 6000 years ago [78]. The shift from oral to written systems marked an important transformation that enabled using our cognitive abilities more systematically. These systems reduce our cognitive constraints in processing and remembering information, making mathematical calculations and building on existing knowledge [21]. Geometry can be traced back to around 5000 years ago in Mesopotamia and Egypt. It involved principles of areas, lengths, angles and volumes that were used for surveying, agriculture, construction and astronomy [79]. Ancient Egyptian and Norte Chico civilizations constructed vast pyramids at least 4500 years ago. This requires – both then and today – applying principles in engineering, architecture and geometry, grounded in systematic measurement, planning and experimentation. An Egyptian medical textbook from about 1600 BCE provides detailed experimental knowledge of dealing with injuries, fractures, tumours and various surgeries and it applies the methods of examining, diagnosing, treating and prognosis [80].

In the Old Testament, Daniel (1: 12–13) [81] describes an experimental trial: ‘Test your servants for ten days. Give us nothing but vegetables to eat and water to drink. Then compare our appearance with that of the young men who eat the royal food [and drink wine], and treat your servants in accordance with what you see.’ Only by combining our cognitive abilities can we – then and now – test such a hypothesis and conceive the design for such a controlled experimentation. We need to apply causal reasoning to test whether a potential cause (a vegetarian, water-based diet) has an effect on people's physical appearance by systematically comparing the outcomes between the two groups after 10 days, and then deriving inferences from the outcomes to modify people's diets in the future. We then combined controlled experimentation with randomisation and blinding in the 19th century which further reduces bias [82].

### 2.3 Cognitive science of science

Our mind makes doing science and creating knowledge possible. It allows for vision needed to make observations, memory to recall what we observe, language to express what we observe, and reason to solve problems and develop scientific methods [22,70]. Our mind shapes how we make sense of our environment, on one hand. On the other, we face cognitive and sensory constraints imposed by nature and evolution, and also constraints to the methods and instruments we develop using our mind. They set the scope within which we are able to create knowledge.

Our cognition is the result of evolutionary processes over the past few million years. This basic fact helps understand our cognitive abilities and limitations: our mind has evolved, within our environmental and cultural niche, in large part in response to problems we have faced up to now [21,45,49,[83], [84], [85]]. Our evolutionary history is one that did not involve observing and mentally modelling phenomena such as the size and nature of the universe, the historical origin of life, global financial markets and the emergence of consciousness. Only in recent history and by creating methods and instruments have we been able to develop such complex theories about phenomena that we do not have sensory experience of and do not directly affect our biological fitness. This has been a great mystery of our mind and science [86]: how have we, given our evolutionary history, evolved the ability to do science and develop elaborate knowledge? We have evolved abilities to observe, solve problems and experiment that enabled developing tools, shelters and agriculture and helped meet our needs. Creating scientific knowledge today is made possible by using these same abilities but for purposes that do not directly influence our survival as when they first developed.

Many phenomena in the world lay beyond the directly observable conditions in which our mind and senses have developed. The further we move away from these conditions – from the surface of the earth and our ecological niche – we require greater abstraction. Our mind is not able, without methods and tools, to access phenomena beyond our senses: atoms and photons at the quantum level, magnetic fields and gravitation, the speed of light, the earth's core and planetary systems, global economic markets and political systems. Our evolved cognitive abilities do not allow us to process large sets of observations or understand highly complex phenomena well. Methods and instruments we have created using our flexible mind explain most of the expansion of science by enabling us to study phenomena that would otherwise lie beyond our cognitive and sensory reach.

Our vision is the most common form of evidence used to explain phenomena. We generally understand phenomena more abstractly as we move from what is observable to non-observable. In physics, we can see this in the difference between Newton's focus on the more easily observable aspects of the world and the less observable world of quantum mechanics which is more difficult to grasp. Because many topics studied by theoretical physicists and theoretical economists are not visible, the challenge of making *sense* of parts of the world is often related to our limited human senses and the ways we can observe phenomena using methods we develop to enhance our senses. This helps explain the constraints we face in collecting data to test and verify different theories in theoretical fields – such as string theory and theories of multiple universes.

### 2.4 Psychology of science

When we do science and acquire knowledge, our mind faces psychological biases. We face limited mental resources, time constraints and incomplete information, so we use simplified heuristics like rules of thumb or shortcuts when reasoning [84,87]. We often rely on existing assumptions and evidence when formulating a hypothesis or applying a method rather than testing them every time. Our mind has largely evolved to absorb and process a limited amount of information and then make quick assumptions and conclusions. We think fast, habit-based and use heuristics most of the time. This can result in unconscious biases [87,88]

A study of ecology scientists showed that they have low awareness and understanding of the importance of their own unconscious biases and how to mitigate them [89]. A study of forensic specialists illustrated that they generally viewed their own judgments as almost infallible [90]. A study of medical doctors illustrated that they regularly made errors in clinical practice due to cognitive biases [91]. Our cognitive constraints can present biases throughout the scientific process. This includes when designing and conducting experiments (such as confirmation bias when searching for evidence consistent with the hypothesis being tested), analysing data (such as omission of some results and poor understanding of statistical methods) and writing up results (such as HARKing bias and confirmation bias when only reviewing literature consistent with the hypothesis) [92].

Our reasoning is also influenced by personality traits [93]. Drive and discipline foster systematic reasoning, just as curiosity and creativity foster how we do science. Intellectual stimulation and recognition can provide motivation to solve a problem or develop a new theory. Goals and needs also influence and motivate us. Personal interest and social contribution can coincide for mutual gain [93,94]. Competition can help ensure quality control and independent testing of others’ work. The sociologist Robert Merton highlights that most renowned academics are known to be driven by a desire for fame – from Galileo, Newton and Faraday to Darwin, Planck and Watson [94].

To better understand science, we need to study not only our individual biological, cognitive and psychological constraints and biases within us – that is, internal factors (discussed in previous sections). We also need to study the collective methods we develop using these abilities and the range of external factors that include social, economic and historical influences (discussed in subsequent sections) (Fig. 1).

### 2.5 Sociology of science

There were hundreds of scientists a few centuries ago [95]. Today, there are about nine million full-time scientists [62]. Demographic growth and complex social organisation have been crucial for the scientific community to grow and for greater collaboration, cumulative knowledge and methodological development. Doing science is thus not just a cognitive activity conducted by individuals but has become an increasingly complex social activity conducted among a community of researchers [21,73,83,96].

The scientific community we are embedded in influences which problems, questions and objectives we find relevant. It shapes which methods we consider credible for analysing them, which experimental designs we choose, the way results are assessed and what assumptions are allowed. It shapes how we classify phenomena and define variables that influence how we measure and understand phenomena. It sets norms such as the kinds of evidence accepted (and not accepted), the types of hypotheses and theories that are suitable (and not suitable), and the forms of peer-review that are appropriate (and inappropriate) [97]. These norms are defined differently across fields and change over time. Together, they account for the rules of the game for doing science.

Robert Merton was a foundational figure in the sociology of science. He argued that science and scientific advances take place within a scientific community with shared scientific norms, values and institutions [98]. He found that renowned scientists receive much more credit for their research than less renowned scientists with equally important contributions (known as the Matthew Effect) [99]. Two other leading sociologists of science Latour and Woolgar observed that scientists within a leading biological laboratory are exposed to peer and social pressures and seek influence. And influence is not just achieved by the theories they develop but by the scope of their social networks and their ability to mobilise support for their work [10,11]. Latour helped develop the actor-network theory that is a constructivist social theory in which everything in the world takes place in changing networks of relationships, with scientific knowledge an outcome of relationships between objects, ideas and humans in scientific practices. In attempting to explain science, Latour and Woolgar argued that social influences are the most important factor in creating knowledge – though they only focus on one factor in studying science, namely social influences. This is a case in point for why we must take the range of cognitive, methodological, demographic and other factors into account when analysing science to avoid misattributing and overvaluing the role of a single factor.

In fact, a complex method (from statistics to randomised controlled experimentation) has not ever been created by an individual mind but by many individuals collectively. An academic field (from molecular biology to nuclear physics) has always been developed by working together cumulatively. A complex theory (from quantum theory to a theory of the origin of life) has not been created by the mind of a single person without relying on much previous knowledge. To create such knowledge we need to acquire and share information cumulatively and collectively.

### 2.6 Economics of science

We can foster science through economies of scale, a reward system, science policy and targeted funding [[25], [26], [27], [28],^40^,^100^]. As society becomes more productive and diversified in providing goods and services, more individuals can dedicate themselves to scientific activities. Larger communities of scientists have a comparative advantage over individual scientists in cumulatively building on research. Science can function like an economy: just as a growing and more specialised labour force generally develops more diversified goods and technologies, a growing and more specialised scientific community generally develops more diversified knowledge and methods. Economies of scale facilitate greater division of labour across and within fields – and thus greater methodological diversity and knowledge.

Science runs on a priority-based reward system that motivates innovation [68]. It gives priority to the first person to publish a new idea or method. It requires making research and methods publicly available [25,28,101]. As a form of intellectual property right, priority is rewarded through social recognition from the scientific community and through potentially contributing to society. This winner-takes-all system incentivises scientists to produce and share knowledge [68,94] and generally more so than monetary incentives [25].

Public institutions help plan, finance and manage how we produce, distribute and use knowledge [97]. They set government priorities and resource allocation – with funding also influenced by overall economic development. Scientists do not generally have large funds to conduct research in areas like cost-intensive basic research or research yielding returns after many years [5,25]. The costs of laboratories and running experiments in some fields within chemistry, biology, medicine and especially physics and astronomy are at times too high for individual researchers.^(ibid.)^ CERN's large hadron collider – the world's largest particle accelerator – cost for example billions of dollars. Much research is however conducted using low-cost methods and instruments, such as statistical and mathematical methods, light microscopes, electrophoresis, assay techniques, chromatography methods and centrifuges.

### 2.7 History of science

Science has a history. Thomas Kuhn, the most-cited historian of science to date, offered an explanation of the history of science that rejected the view of scientific change as being cumulative [1,32]. The history of science can be viewed as a cycle in which established ideas and facts are doubted, new problems and evidence then lead to new revolutionary ideas and facts, which eventually over time are also doubted once problems and anomalies associated with them become apparent, and the cycle begins again. For Kuhn, this process is not cumulative but reflects revolutionary paradigm shifts, in which a scientific community rejects existing assumptions and theories and adopts entirely new ones. This notion of science may seem to apply to shifts in physics in the past, namely shifts in theories of physical reality from Aristotle to Newton to Einstein. Kuhn focused on such cases largely in physics up to the early 20th century [1]. The shift from the Ptolemaic earth-centred theory of the universe to the Copernican sun-centred theory characterised the classic paradigm change, which Kuhn focused much research on.

Yet no major scientific methods used across fields (such as statistics, x-ray methods or controlled experimentation) and no major scientific fields that produce reliable knowledge (such as biology, chemistry, nuclear physics and computer science) have been entirely discarded. Rather, we cumulatively extend them over time. Our scientific methods and fields encompass our extensive bodies of knowledge consolidated over time. Shifting our attention from individual hypotheses and select theoretical discoveries to all major scientific discoveries, methods and fields is the best way to measure and assess the cumulative nature of science. For they make up the foundation of science and how we conduct science and they encompass our established bodies of knowledge.

Science is cumulative and scientific theories and methods are not independent of their historical context. They are provisional and have been expanded by new evidence, experiments and methodological advances over time. There is a history of science – not just one science that is constant over time.

### 2.8 Anthropology of science

Anthropology of science is the cross-cultural study of humanity which retraces how we have developed science, from the past to the present. We developed increasingly complex language an estimated 50,000 to 100,000 years ago [78] that has enabled explaining our observations and ideas to others and thus enabled greater cooperation and tool-making. Our expanding cognitive and social abilities gave us an increasing advantage, likely for the first time, over many other smart animals. We created measurement tools like simple tally mark systems at least 35,000 years ago, and eventually agriculture (an estimated 12,000–13,000 years ago) [78] that enabled increasing our food and labour productivity. We could increasingly dedicate our time to other cognitive activities, beyond meeting our basic needs [78]. As villages expanded into cities and cities into empires several thousand years ago, growing populations were able to build on cumulative knowledge, increasingly specialise and further develop written language and complex numerical systems – two essential features of contemporary science [78]. Some cultures maintained a degree of stability over centuries, and even millennia, such as the ancient Chinese and Greeks [54]. Such stability allowed building extensively on what we know. Population density, specialisation and methodological diversity increase together, with changes in one generally affecting the others. Scholars especially in ancient China and Greece studied a broader range of phenomena, from astronomical events and the properties of living animals, to magnetism and sound. They did so with a logical view of how the world is broadly construed and viewed certain phenomena as operating according to general principles [54,102]. Using a pragmatic experimental approach, ancient Chinese developed many more advancements than ancient Greeks: effective immunization techniques, magnetic compasses, negative numbers and the ‘Pascal’ triangle, astronomical observations of novae, seismographs, irrigation systems and quantitative cartography, as well as papermaking and printing that fostered the spread of knowledge [54,102,103]. For ancient Chinese to develop smallpox vaccines for example required (just as today) complex understanding of the causes and effects of infectious disease, their interactions and how to control them [102]. Because the Chinese created a complex system of astronomical records (including star catalogues and observations of eclipses and novae) our records today are able to go back millennia [102,104]. In the centuries leading to the 1500s, we exchanged technologies more rapidly and eventually globally [78]. We had already widely used systematic observation, measurement and experimentation to develop increasingly sophisticated technologies: eyeglasses, windmills and mechanical clocks in 13th century Europe and the microscope in the 16th century [105]. Eventually, these methods and instruments were applied not only to questions whose practical relevance was directly observable (technological knowledge) but also not always directly observable (purely scientific knowledge). What made the work of 17th century scholars like Copernicus, Galileo, Boyle and Newton possible is a cumulative process of greater technological advances and greater awareness of these more systematic methods already widely used. They also commonly used the newly developed instruments including microscopes, barometers and telescopes that made many of the discoveries possible. Overall, scholars at the time expanded science by combining our evolved methodological abilities and adopting written language, mathematical systems including geometry and algebra, and diverse technological and scientific knowledge developed by our ancestors over thousands of years. These are the historical hallmarks in the development of our human mind, social organisation and methods that have enabled developing science. Science is thus not just a product of 17th century Europe.

### 2.9 Methodology of science

Over our species’ history, we evolved methodological abilities of the mind (observation, problem solving and experimentation) that we use together with increasingly complex methods developed using these abilities (controlled experimentation, statistics and x-ray methods). Science has always been grounded in these evolved methodological abilities (our *universal* methodological toolbox) that have enabled developing vast bodies of knowledge by creating sophisticated methods (our *adaptive* methodological toolbox). As we face constraints using our evolved abilities, we have developed methods and instruments to reduce them. Such constraints are cognitive (such as limited cognitive bandwidth and memory), social (such as cultural values, norms and interests), geographic (such as differences across contexts that require conducting studies in multiple contexts) and so forth. Importantly, we develop new methods and instruments that enable us to better access and understand the world and make new advances by addressing our human and methodological constraints. Mathematical methods are used across fields to help us systematically calculate and measure phenomena and represent them using algebraic equations. Controlled experimentation and randomisation are used in fields from biomedicine to psychology to reduce biases in designing, implementing and analysing studies. Magnetic resonance imaging enables detecting phenomena like magnetic fields and radio waves that we do not have sensory receptors for. Electron microscopes enable perceiving miniscule objects using the wavelength of an electron.

Methods and instruments can however bring constraints and possible biases in developing knowledge. Each mathematical technique, x-ray method and statistical method generally has limits as to which questions we can study and what conclusions we can derive using it. Each has a set scope within which we can capture or model phenomena, design, implement and evaluate experiments, and interpret results. Each requires making assumptions. One of the best ways to reduce individual methodological constraints is by applying multiple methods [38,106]. For each method can provide different evidence and perspectives into a phenomenon. Each method can confirm whether results consistently point in the same direction. In sum, our mind's methodological abilities and complex methods and instruments we create using these abilities are at the centre of understanding science.

### 2.10 Scientometrics and network science

Science describes and explains the world through research articles and books that are organised into scientific fields. Scientometricians including network scientists analyse this scientific literature. They rely on the indicators of citations and publication counts to study issues such as research productivity, team collaborations, career dynamics, networks of scientists and institutions, and novelty [4,5,8,9,33,36,[107], [108], [109]]. They use the methods of large-scale data (big data) and network analysis and search for patterns in such data.

Studies on innovation illustrate that researchers are generally risk-averse, choosing to study phenomena in which they already have expertise. This limits what is studied in the future and making potential new discoveries. Researchers willing to explore new areas and undertake a riskier career, moving from traditional topics to riskier innovation, are more likely to expand a field and make discoveries. What characterises high-impact science are conventional combinations of existing work that integrates novel combinations of not-yet-connected topics [110] or research methods. To increase the impact of research, scientists need to show how it contributes to established research [111].

In terms of researcher productivity and impact, major discoveries are generally made by younger researchers and explained by their higher productivity and not yet securing permanent positions [4,7]. Studies generally illustrate a median age of discoverers between their mid-30s and mid-40s [60,112,113]. There has also been a general shift from slightly over 1 to about 5 individuals per team in science and engineering between 1900 and the early 2000s [114,115].

Moreover, most measures of scientific impact and success use citation counts. Yet measuring success using citations can constrain us in developing new ideas since it gives an advantage for highly cited researchers to become even more cited. It also provides a bias for researchers to use more cited and thus at times older research. It also disadvantages younger researchers and innovative researchers working between disciplines and paradigms [33,99]. Scientific institutions need to place greater focus on other metrics of success beyond citations, such as levels of innovation and societal relevance of research [5] and the development of new methods and instruments. Citation counts do not capture the immediate impact of new ideas or breakthroughs in science (as citations take time to accrue), the impact on policy or society (as they cannot be cited) or the powerful role of scientific methods and instruments (as they are not always cited).

### 2.11 Computer science of science

We are constrained by our limited cognitive and computing capacity when studying the world. We are flooded with vast new data and publications each year, at a pace far exceeding our human abilities to process the influx of information and data. Computers play a central role in science and studying science by expanding our cognitive resources, memory and capacity for data processing, analysis and simulations [41]. We now often have almost immediate access to the vast range of existing methods and bodies of knowledge in science.

A critical bottleneck in making computers and the internet possible was overcome in a landmark article *A mathematical theory of communication* in 1948, in which Shannon conceived the digital nature of information as binary digits (0 or 1). This completely shaped how we began to use computational data. Today, data are widely used as binary digits across science, for example in statistical analysis to capture phenomena in the form of variables [116]. By demonstrating how we can quantify digital information, Shannon's work has been called the Magna Carta of the digital age [117].

A rapid increase in computing power and available data has also accelerated growth in artificial intelligence [41,118]. Machine learning applies computer algorithms that improve automatically through an iterative process of using a given dataset. It enables delegating some aspects of data collection and analysis to automated computer programmes. This is especially relevant when we study phenomena for which we have vast data or require making quick decisions. In biomedicine for example, methods exist for drug design that automate many mechanical tasks performed by researchers. Researchers provide the collected data that are coded and inputted into robotic platforms that conduct a series of experiments and generate results [43]. These new methods can complement (not replace) human expertise [42,119].

Computer scientists have also offered a computational account of discovery by attempting to simulate discovery processes [[120], [121], [122], [123]]. Computational accounts have been ambitious, attempting to develop algorithms that could drive new discoveries. But they have mostly only focused on the path from data to scientific laws, and do not analyse the role of methods and broader background factors in the discovery process that are taken as given. To date, they have had only limited success in reproducing past discoveries and do so at a high level of abstraction [120,124]

### 2.12 Statistics and mathematics of science

Revolutions are easy to spot when they happen as a single extraordinary event. The impact of some methodological discoveries, such as x-ray analysis in 1895 or the gene editing method CRISPR in 2012, was immediately known around the world. Other methodological discoveries are difficult to spot, as they take place over centuries. The development of mathematical and statistical methods reflects such slow methodological revolutions that transformed how we do science and how we understand the world. Statistics and mathematics are arguably the two most widely used methods across science. In physics, the field's two central theories are quantum theory (which incorporates probabilities and exhibits indeterministic behaviour) and relativity theory (which is described by mathematical formulas and is deterministic). In many fields of science, inferential statistics has become synonymous with the scientific method.

Modern statistics transformed science by allowing us to study the world with vast amounts of data in more complex ways and conduct larger-scale experiments. We apply statistical methods to study basically any phenomenon in science, from cells and viruses in populations, to planets, economic markets and science itself.

Because science relies heavily on statistical methods, the quality and replicability of our evidence also depends on how we design statistical studies, refine statistical methods and report statistical results. Yet there are common problems in producing reliable and replicable scientific results, including small sample size and low statistical power [125], p-hacking and selective reporting, small effect sizes and HARKing [29,38,[126], [127], [128], [129]]. Small sample size and low statistical power can affect studies negatively by increasing the chance of false positive results. *P*-hacking occurs when researchers for example collect additional data after assessing the statistical significance of results or exclude outliers to improve statistical significance. It arises given pressure to report only statistically significant or positive results, since journals are less likely to publish studies with statistically insignificant or negative results (publication bias). Such issues often lead to statistical biases in studies, including sampling and measurement bias [29,106,[125], [126], [127]], and have contributed to a replication crisis in science. Just as the replication crisis is driven by methodological, structural and psychological causes [130], science in general is also driven by a broad range of factors.

### 2.13 Philosophy of science

What science is and its foundation have been explored by philosophers for centuries including Bacon, Hume and Popper, and what knowledge is and its foundation for over two and a half millennia including Plato, Aristotle and Wittgenstein. Philosophers have addressed central questions of science of science longest. Major debates that have dominated philosophy of science include paradigm shifts, justification, induction, demarcation and realism. ^(18,19,185,186,187)^ Paradigm shifts refer to fundamental changes in theories [1]. Justification deals with principles such as falsification and verification to justify our theories [15,131,132]. Induction addresses the question of whether observations we make can or cannot justify generalising about the observations in other contexts or in the future [133,134]. Demarcation involves defining criteria for what is and is not science [15,16,98,135]. Realism concerns whether theories provide a reliable approximation of reality, for observable and not directly observable phenomena [136,137]. This discipline also covers domains such as philosophy of physics, biology and economics that provide insights into the complexities of these fields’ concepts, definitions and assumptions.

Explaining how we create knowledge and how science operates are in philosophy at times reduced to two theories of scientific methodology: induction and falsification. Bacon's theory of induction and scientific methods is commonly viewed as the conceptual origin of modern science, namely using the methods of observation, experimentation and deriving conclusions [138]. These are the same methodological abilities of the mind that our species have always used to acquire knowledge. In general, inductive reasoning is when we for example repeatedly observe the sun rising, and then infer that it will rise tomorrow or will always rise. It allows going beyond our current set of observations and drawing conclusions about the future [133,134,138]. For falsificationists in contrast, scientists need to do science by constructing hypotheses and theories, testing and attempting to falsify them [14,15,139]. Popper argues that the defining trait of scientific investigation is the principle of falsification – which is the most influential account in the philosophical literature [139]. Scientists have not adopted falsification as a guiding principle for evaluating theories. For both, theories like Freud's psychoanalytic theory are not scientific as we cannot easily test or falsify them.

When we describe regularities in the world there is also often a trade-off between simplicity and strength: the greater the simplicity used to describe phenomena, the greater the loss in power to explain them [140]. The best explanation, theory or ‘law’ would ideally account for strength and simplicity. Newtonian physics for example has less strength but is simpler than quantum physics.

Overall, philosophy of science has not yet provided a comprehensive understanding of its subject matter – science. For most articles have focused on studying theories (an output) [18,19,[141], [142], [143]] and not on the process of science, especially scientific instruments and methods such as x-ray methods, computational methods and particle accelerators. Most philosophers have studied what science is using theoretical and normative approaches and using their mind as the main source of knowledge, rather than also using experimental and statistical methods. Most articles are also commonly an internal response to philosophical questions but not always to problems facing science. ^(ibid.)^

### 2.14 Linguistics of science

Without a system of language we would not be able to reason complexly, express our knowledge and do science. It enables explaining to others what we observe, how we solve problems and the knowledge we acquire [21,78,144]. With language we can quickly obtain and pass along methods and bodies of knowledge. How we use language determines how studies are expressed and disseminated and how accessible they are to researchers in the same and other fields.^(ibid.)^ [49,83]

Written and especially digital documentation enables more efficiently storing, sharing and building on vast bodies of knowledge and methods across generations [21]. A system of written language is a precondition for cumulative knowledge and creating scientific methods [145]. Only by using language can we express our methods of science, including statistical coefficients and algebraic equations. Moreover, technical language divides the scientific community. A specialised language connects researchers in one subfield with a common language but often presents a barrier for researchers in other subfields.

At present the English language dominates science worldwide, including journals and institutions. Most studies across science of science generally only study articles in English and so can provide an incomplete picture of science. Language and writing systems – like scientific methods – are also thinking tools. The Western alphabet is viewed as structured analytically and a natural tool for categorising. It functions as a model for classification systems, and standard measures and weights. The Chinese writing system, on the other hand, is largely pictographic and non-reductionist. It functions as a model for viewing the world as continuous and holistic [54,103]. Using a particular alphabet can shape the way we think and view phenomena.^(ibid.)^

How science is communicated to the public, policymakers and scientists is also important as it can affect their decisions [146]. Studies on climate change, nutrition, vaccinations and the coronavirus are relevant to the general population. Scientists need to ensure that a study's results cannot be easily misinterpreted, political and ethical aspects are presented sensitively and uncertainty and values are communicated in a balanced way – as they can negatively affect our choices [147]. Language used in social media, news outlets and popular science books can be susceptible to misuse, as they do not undergo rigorous peer-review but can have a broad impact [146].

## 3 Science of science: an integrated and methods-driven understanding of science

We have developed science by using our cognitive and sensory abilities that have evolved within our environmental niche of the world and they thus face constraints, and we expand our vast knowledge of the world by developing new scientific methods and instruments designed to reduce our constraints. This universal and adaptive methodological toolbox of ours is at the centre of science and enables us to do and advance science in new ways. Other factors also influence science as we are social beings embedded in our scientific community and its practices, socialised into a system of language and mathematics used to express our knowledge, born into a historical context with particular worldviews, abiding by scientific norms, principles and assumptions, motivated by biological traits, subject to psychological biases, aided by computer technology, and influenced by recognition and ambition, funding and societal objectives, public and economic institutions. Ultimately, science is a dynamic system of human activities aimed at better understanding the world. What drives this system are complex interactions between our evolved mind and the methods we develop using our mind, on one hand, and the world and social institutions, on the other. Science is the outcome of arguably the most cognitive and social activity that our species has undertaken.

Scientists are members of a scientific community shaped by its methodology, its history, its sociology and its philosophy. What were once thought of as independent disciplines – with tens of thousands of publications studying science from their own disciplinary perspective (Fig. 2) – have been linked together here to account for science holistically (Fig. 1). To address questions about science, some view the appropriate unit of analysis to be the individual (psychologists and cognitive scientists), the group (anthropologists, sociologists, economists and linguists), the species (biologists and evolutionary cognitive scientists), the past (historians, archaeologists and some anthropologists) or the meta-level or methodological level (methodologists, scientometricians, computer scientists, statisticians and philosophers). This common approach to studying science has led leading researchers to not yet address the central questions of how important the particular ‘key’ factor they study is and how it relates to other ‘key’ influencing factors identified by other researchers. Different researchers working at a different level and on a different aspect of science of science are driven by the common aim of understanding and improving science. But they thus at times do so in methodological and disciplinary silos that has led to a fragmented understanding of science.^(ibid.)^

The unified account of the field of *science of science* presented here, by bringing together the approaches and features across 14 disciplines, outlines the evidence on science that is coherent across the natural, behavioural and social sciences. Taking such a holistic approach represents the most comprehensive understanding we have of science for the following reason: *the range of disciplinary approaches apply different methods and focus on different features of science, and there is coherence across the independent strands of evidence, in particular in the role of methodological features in shaping science*.

We cannot comprehend the individual factors in isolation, because the parts (and the groups) interact with each other to account for the greater whole. The common approach of studying science from one perspective would be like trying to explain an ecosystem by only studying trees, or the human body by only studying cells. We learn a lot, but that knowledge remains incomplete.

**Appendix Fig. 1:**
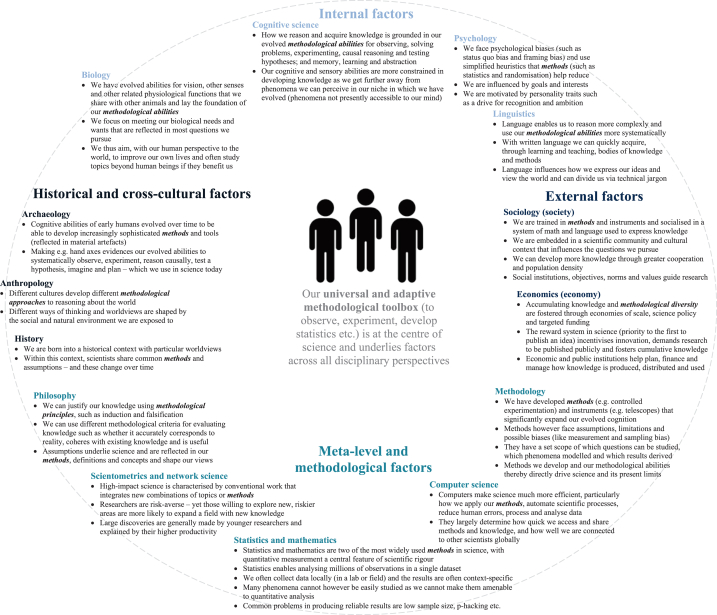
The origins, foundations and limits of science: the range of interconnected factors, and particularly our methodological toolbox, that enable and constrain science Note: Other influencing factors can be included for example into economics of science (such as the political usefulness of research) or into philosophy of science (such as ethical issues limiting what topics we can research and what experiments we can conduct). Regulatory bodies for instance place limitations on research related to human cloning, aspects of gene editing and technologies fostering climate change. We need to view the classification across the four areas loosely, with connections taking place across factors and areas.

Overall, there is no consensus among researchers on which proposed explanation of what drives science is best. Scientific consensus is however a central feature of science. A central advantage of integrating the various factors is that it enables uncovering which are most important and what central factor is shared in common across the different fields. We find here that our methodological toolbox underpins the different factors across all disciplinary perspectives and is the only factor that does so. A simplified summary of the set of interconnected abilities and conditions that enable and constrain science is provided in Appendix Figure 1 (with *methodological features* placed in italics). The degree to which other factors influence our scientific advances and discoveries varies depending on the phenomenon we study. This integrated approach thus enables developing a more coherent understanding of science and grounds the new-methods-drive-science theory: no factor plays as foundational and ubiquitous a role in understanding the origins, foundations and limits of science as new scientific methods and instruments we develop using our mind's methodological abilities (Appendix Figure 1).

No other factor influencing science is relevant in all fields – with for example linguistics and archaeology providing little, if any, insights into commonly mentioned factors like scientific funding, incentive structures and the scientific community, and also new theories. In ground-breaking scientific publications, we observe that teams can be small or large, low or high funded, young or old, at low and top ranked universities, or interdisciplinary or not. Money, collaborations and a research community are basic factors that foster science, but alone are not enough to break new ground.

This account of science explains how our scientific methods and instruments and our human mind used to develop them set the scope within which we are able to develop knowledge and science. Then, beyond nature and our cognitive and methodological limitations in understanding nature, influences that are economic, social, historical and the like, even if often less direct and important, also shape the content and scope of the knowledge we create. We need to place our scientific methods at the centre of focus while studying this broader range of factors and how they interact with our methods.

**Appendix Fig. 2:**
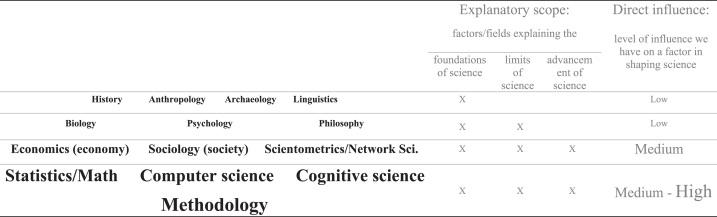
Scope of factors/fields in explaining and shaping the foundations, limits and advancement of science

We can depict the level of scope that a given factor has in explaining science, and the direct influence we have on that factor in shaping science. These are the two criteria used to assess each factor studied here: we find that our scientific methods and instruments have the greatest explanatory scope and direct influence in science – see Appendix Figure 2.

## 4 Science of science: an integrated field grounded in the new-methods-drive-science theory

We offer here a foundation for the integrated field of *science of science* that studies science, and its foundations and limits, by combining methods and evidence from across the sciences. Establishing the field of *science of science* requires providing not only an empirical foundation but also a theoretical foundation for understanding science. The *new-methods-drive-science theory* presented here can offer a unifying theory for the field that is grounded in the powerful role of scientific methods which is the common thread among this scientific community. The theory can integrate the disparate fields studying science as our methods and instruments are connected to all features of science (see Appendix Figure 1). Our evolved methodological abilities of the mind (our *universal* methodological toolbox) and sophisticated methods and instruments we develop using our mind (our *adaptive* methodological toolbox) are what directly enables us to develop knowledge and science. Our scientific tools allow us to do science and also set the present limits of what science we are able to do. The theory describes how our methods have driven the origins, foundations and present limits of science.

The *new-methods-drive-science theory* explains how we advance science by developing new methods or refining existing methods that expand our present cognitive, sensory and methodological reach to the world. New methods and instruments – such as novel statistical techniques, x-ray methods and telescopes – enable making new breakthroughs by reducing our present constraints to studying the world in new ways. In contrast, existing leading (competing) accounts of science are outlined throughout the paper – for example in the history of science by Kuhn who argues that science goes through paradigm shifts in theories [1,32]; in scientometrics in which scientists argue that career trajectories, team collaboration, research output and networks of scientists are the central parameters driving science [4,5,9,33,35,36]; in the sociology of science by Merton [98] and Latour and Woolgar [10] who highlight the central role of social factors shaping science etc.

In describing the new-methods-drive-science theory, we define the central terms here. *Science* is the study of the natural and social world by using our cognitive abilities (including observation, experimentation and problem solving) and the methods and instruments we develop (including statistical techniques and algebra, and particle accelerators and electrophoresis) with the aim of describing, explaining, predicting and controlling phenomena (as outlined earlier). Scientific *methods* are systematic techniques and scientific *instruments* are systematic tools used for scientific research and which are generalisable (and do not include other features of science such as concepts, theories and language). In general, if more scientific methods are created, then more scientific progress will be achieved. An assumption of the theory is that basic factors are in place, including our cognitive abilities and a minimal level of funding and collaboration to generate methods and tools.

The theory connects our new scientific tools to scientific progress. For they are what allow us to observe farther, process information better and measure phenomena more precisely, providing new perspectives to the world. Our methods and instruments are how we experiment with and control different phenomena in the world and expand our scientific scope. They determine how we design, implement and analyse studies and how we define and gather evidence. We can better understand science in light of this integrated *new-methods-drive-science theory*. This theory:

• places us, and the methods we develop using our mind, at the centre of studying science;
• integrates evidence of the abilities and conditions that have enabled developing science (biological, cognitive, social and methodological), the abilities and conditions shaping the scope of science (including, in addition, historical, economic etc.) and, most importantly, the abilities and conditions allowing us to expand the present limits of science (mainly methodological but also cognitive, sensory and social);
• combines thus insights into the origins and foundations of science (especially our evolved methodological abilities of the mind and the methods we develop) with insights into the present boundaries of science and how to push them (especially addressing our methodological constraints);
• pools together the range of methods used to study science to provide an integrated explanation that is consistent and better grounded in what is already known across disciplines.

Because our methods are at the centre of how we do science and make discoveries, understanding the foundation of the methods we develop and use is at the centre of *science of science*. The fundamental importance of our methods is evident for all aspects of science: conducting, evaluating and advancing science and also understanding science. The theory predicts that scientific progress will be brought about by generating novel methods, and the theory can be directly tested. ^see^ [61,148] In terms of the scope of the theory, it applies across the natural and social sciences.

## 5 Conclusion

Taking an integrated perspective to the field of *science of science* can offer answers to fundamental questions about science: its origins, evolution, foundations and constraints. Integrating diverse knowledge across diverse disciplines using diverse methods into a holistic field has been the central challenge of the field of *science of science*. A holistic framework enables filing down the often inflated role of a single factor and evaluating which factors are most important and how they fit together, to then be able to develop a more coherent understanding of science. Here we outlined the powerful role of our *universal and adaptive methodological toolbox* in driving science. We observed that the central factors proposed as the most important factor explaining science – namely paradigm shifts in theories [1], the principle of falsification of theories [14,15,139], social influences on scientists [10,11] and so on – do not have as much explanatory power and direct influence on the foundations, limits and advancement of science compared to other factors (Appendix Figure 2). These factors proposed by the most cited researcher within specific fields – namely Kuhn in history of science, Popper in philosophy of science, Latour and Woolgar in sociology of science and so on – need to be left in the background. Our methodological toolbox needs to be brought into the forefront of how we understand, study and advance science.

What are the benefits of integrating these fields into a holistic science of science that the economist of science, scientometrician, philosopher of science and other researchers did not have beforehand? For some researchers it is a shift towards a joint research focus on methodology and better understanding and improving our best methods and instruments that drive science and discovery. For other researchers it is addressing the fractured disciplinary approaches and explanations about an often overemphasised role of power by sociologists, citations by scientometricians etc. to develop more integrated explanations. For all researchers it is a shift to studying science in an integrated way, coherent with already common knowledge in other subfields, rather than in isolation.

The origins, foundations and limits of science can be better explained and advanced in light of the *new-methods-drive-science* theory. Our methods and instruments are the only factor that underpins all fourteen subfields and that we are most directly able to influence to do and advance science. A central focus on scientific methods across all subfields is also crucial to the integration of science of science.

Constraints of such qualitative studies (that integrate insights from across disparate fields) however include quantitively measuring factors studied across very different fields (norms, institutions, practices, assumptions etc.), balancing more breadth across more fields with inevitably less depth on any single field, and synthesizing across fields as each field has its own methodological preferences and complexities.

Different implications emerge from this meta-approach to studying science. First, measuring the success of the field of science of science can be done by establishing a society, journals, conferences and interdisciplinary institutes at universities that adopt a truly integrated approach to studying science. Scientific institutions (the EC, NSF and other funding agencies) need to begin incentivising integrated meta-scientific research and new methodological research that is as important as other established funding areas in advancing science and discoveries. Second, better training researchers studying science and conducting research more interdisciplinarily is also essential. Disciplinary specialisation in studying a phenomenon as multidisciplinary as science is why we, to date, have not yet developed a coherent general theory of how science advances – the central question in science of science. We would otherwise not even know that we have gained a coherent understanding of science, unless we compare the findings to assess if they are coherent across the subfields of science of science. Third, we can mitigate the set of constraints we face in advancing science by better understanding those constraints. We can mitigate constraints and biases facing our methods by applying multiple methods and developing new methods, reduce social influences by conducting studies in different contexts, address psychological biases by automating some processes using computers, and so on. Essential to improving science is an awareness of the importance of developing new methods and instruments designed to reduce our constraints to studying the world. Fourth, we need to revisit our best methods for assessing and measuring science and discoveries and adopt a broader set of empirical methods from neighbouring fields [37]. Researchers studying science (including scientometricians, network scientists and economists of science) commonly study one factor descriptively, i.e. conduct descriptive studies using observational data. Such studies can uncover important relationships. But a critical step to move the field forward will be conducting more studies that can assess causal effects of factors driving science – i.e. turning to established methods such as in economics and medicine. We need to conduct longitudinal studies that follow scientists to assess changes over their careers: including the effects of discoveries, new collaborations and relocating to top universities. We will have to begin applying a wider range of methods: instrumental variable methods, natural experiments, randomised controlled experiments, institutional analyses, and so forth. A comprehensive and more nuanced discussion of the topic of this paper is the subject of my forthcoming book, *Science of Science: Understanding the Foundations and Limits of Science from an Interdisciplinary Perspective*, to be published by Oxford University Press [149].

Ultimately, a unified field of *science of science* holds a vast potential for tackling foundational questions about the origins, foundations and limits of science. It holds a vast potential for better understanding how we drive new scientific advances and methods that open new and unknown frontiers.

## Funding

I am grateful for funding received by the 10.13039/501100000780European Commission (Marie Curie programme) and the Ministry of Science and Innovation of the Government of Spain (grant RYC2020-029424-I, and PID2021-126200NB-I00).

## Data

No primary data were used for the research in the article but rather secondary literature.

## Ethics approval

Approval from an ethics committee was not required because no data for participants nor sensitive information were included.

## Informed consent

No informed consent was required.

## Declaration of competing interest

The author declares that he has no competing financial interests or personal relationships that could have appeared to influence the work reported in this paper.

## Appendix

### Scope of factors/fields in explaining and shaping the foundations, limits and advancement of science

We depict here the level of scope that a given factor has in explaining science, and the direct influence we have on that factor in shaping science. These are the two criteria used to assess each factor. Some factors (and fields) mainly only help explain and shape the foundations of science (historical, anthropological, archaeological and linguistic factors) and others can also do so for the limits of science (biological, psychological and philosophical factors). Though, we cannot directly influence them to promote science. These factors are thus classified as having lower scope to explain and influence science. Other factors (and fields), such as the economy, society and scientometric features, help explain and shape the foundations, limits and advancement of science. And we can partially influence them to foster science. These factors thus have medium scope to explain and influence science. Finally, other factors (and fields), such as methodology, statistics/mathematics, computer technology and cognition, help explain and shape the foundations, limits and advancement of science. And we can most directly influence them to make new scientific advances. These factors thus have high scope to explain and directly influence science.

### Calculations of the share and number of publications across the subfields of science

A brief clarification is provided here for how the share and number of publications across the subfields of science of science have been calculated in Fig. 2. Publications, which include articles, books and other scientific formats, were identified by searching a given term in the title, abstract or keywords (Scopus' default search function). Data were collected for the fields that contribute to understanding the origins, foundations or limits of science; data reflect the outcome of searches in early 2024 using the terms ‘biology of science’ or ‘philosophy of biology’ (656 publications), ‘cognitive science of science’ or ‘philosophy of cognition/mind’ (3084), ‘psychology of science’ or ‘philosophy of psychology’ (456), ‘linguistics of science’ or ‘language of science’ (638), ‘sociology of science’ or ‘philosophy of sociology’ (1604), ‘economics of science’ or ‘philosophy of economics’ (319), ‘(scientific) methodology of science’ or ‘philosophy of (scientific) methodology’ (300), ‘computer science of science’ or ‘philosophy of computer science’ (50), ‘statistics/mathematics of science’ or ‘philosophy of statistics/mathematics’ (1225), ‘scientometrics’ or ‘network science of science’ (5418), ‘philosophy of science’ or ‘metaphysics/ontology/epistemology of science’ (10,524), ‘anthropology of science’ or ‘philosophy of anthropology’ (137), ‘history of science’ (8268), ‘archaeology of science’ or ‘philosophy of archaeology’ (23), and ‘science of science’, ‘metascience’ or ‘metaresearch’ (956).
